# A novel integrated care concept (NICC) versus standard care in the treatment of chronic cardiovascular diseases: protocol for the randomized controlled trial CardioCare MV

**DOI:** 10.1186/s13063-018-2502-1

**Published:** 2018-02-20

**Authors:** Christian Schmidt, Alper Öner, Miriam Mann, Katja Krockenberger, Melanie Abbondanzieri, Bernard Brandewiede, Armin Brüge, Gisela Hostenkamp, Axel Kaiser, Henriette Neumeyer, Andreas Ziegler

**Affiliations:** 1 Universitätsmedizin Rostock, Ernst-Heydemann-Str. 8, 18057 Rostock, Germany; 2 Abteilung Kardiologie, Universitätsmedizin Rostock, Ernst-Heydemann-Str. 8, 18057 Rostock, Germany; 3 Amedon GmbH, Willy-Brand-Allee 31c, 23554 Lübeck, Germany; 4 Gesellschaft für Netzwerk- und Innovationsmanagement der Industrie, Bruchstraße 1, 14806 Bad Belzig, Germany; 5 Philips Medizin Systeme Böblingen GmbH, Hewlett-Packard-Straße 2, 71034 Böblingen, Germany; 6 Philips GmbH Market DACH, Healthcare Informatics Solutions and Services, Röntgenstraße 22, 22335 Hamburg, Germany; 7 Lohfert & Lohfert AG, Rothenbaumchaussee 76, 20148 Hamburg, Germany; 8 StatSol, Moenring 2, 23560 Lübeck, Germany; 9 School of Mathematics, Statistics and Computer Science, University of KwaZulu-Natal, Pietermaritzburg, South Africa

**Keywords:** Atrial fibrillation, Care center, Disease management program, Evidence-based care, Heart failure, Hospitalization, Integrated care, Randomized controlled trial, Telemedicine, Treatment-resistant hypertension

## Abstract

**Background:**

Cardiovascular diseases are the major cause of death globally and represent a major economic burden on health care systems. Positive effects of disease management programs have been shown for patients with heart failure (HF). Remote monitoring and telemonitoring with active intervention are beneficial in atrial fibrillation (AF) and therapy-resistant hypertension (TRH), respectively. For these patients, we have developed a novel integrated care concept (NICC) which combines telemedicine with intensive support by a care center, including a call center, an integrated care network including inpatient and outpatient care providers and guideline therapy for patients.

**Methods:**

The aim of the study is to demonstrate the superiority of NICC over guideline therapy alone. The trial is designed as open-label, bi-center, parallel-group design with two groups and a blinded observer. Patients will be included if they are either inpatients or if they are referred to the outpatient clinic of the hospitals by their treating physician. Randomization will be done individually with stratification by cardiovascular disease (AF, HF, TRH), center and admission type. Primary endpoints are based on the 1-year observation period after randomization. The first primary endpoint is the composite endpoint consisting of mortality, stroke and myocardial infarction. The number of hospitalizations form the second primary endpoint. The third primary endpoint is identical to the first primary endpoint plus cardiac decompensation. Adjustments for multiple testing are done using a fall-back strategy. Secondary endpoints include patient adherence, health care costs, quality of life, and safety. A sample size of 2930 gives 80% power at the two-sided 2.5% test level for the first primary endpoint. The power for the second primary endpoint is 99.8% at this sample size, and it is 80% with 1086 patients.

**Discussion:**

This study will inform care providers whether quality of care can be improved by an integrated care concept providing telemedicine through a round-the-clock call center approach. We expect that cost of the NICC will be lower than standard care because of reduced hospitalizations. If the study has a positive result, NICC is planned to be immediately rolled out in the federal state of Mecklenburg-West Pomerania and other federal states in Germany. The trial will also guide additional research to disentangle the effects of this complex intervention.

**Trial registration:**

DRKS, ID: DRKS00013124. Registered on 5 October 2017;

ClinicalTrials.gov, ID: NCT03317951. Registered on 17 October 2017.

**Electronic supplementary material:**

The online version of this article (10.1186/s13063-018-2502-1) contains supplementary material, which is available to authorized users.

## Background

Cardiovascular diseases (CVDs) are the major cause of death globally. An estimated 17.7 million people died from CVDs in 2015, representing almost a third of all deaths worldwide [1]. CVDs are a major economic burden on health care systems in terms of both direct costs, such as hospitalizations, physician visits and drugs, and indirect costs associated with mortality and morbidity, such as losses of productivity due to premature mortality and short- or long-term disability [2]. According to the 2015 German Federal Health Report the costs caused by CVD amounted to 36.9 billion EUR. The total expenditure of healthcare provision for CVDs thus is approximately one sixth [3]. Hypertension alone was estimated to cost 12.6% of USA health care expenditures [2].

People with a CVD or who are at high cardiovascular risk due to the presence of one or more risk factors, such as obesity or diabetes, need early detection and management using counselling and medicines to reduce morbidity and mortality [1]. The success of these strategies depends on patient compliance, which is affected by many factors such as the patient’s knowledge, confidence in ability to follow recommended behaviors, perception of health and benefits of therapy or behavior, availability of social support, complexity of the regimen and the patient relationship and communication with the health care provider [4]. To remediate poor patient compliance the most promising strategies are complex interventions, i.e., combinations of interventions including patient education, self-monitoring, social support, telephone follow-up and tailoring with multicomponent strategies [4].

### The need for improving patient care in Mecklenburg-West Pomerania

Mecklenburg-West Pomerania is a federal state in northern Germany. It is the least densely populated and least industrialized German state, being the sixth largest in size, but only the 14th in population, and it is called Germany’s most rural state [5]. Morbidity and mortality from CVDs was the fourth highest among all German federal states according to the Heart Report 2015 [6]. By using combinations of interventions, we aim at reducing CVD morbidity and mortality. We have selected three cardiovascular diseases, atrial fibrillation (AF), heart failure (HF) and therapy-resistant hypertension (TRH) for investigating the efficacy of our strategy.

### Target diseases: atrial fibrillation, heart failure and therapy-resistant hypertension

AF is the most common clinically important arrhythmia [7]. The estimated number of patients in Germany suffering from AF is 1.8 million, which corresponds to about 2.2% of the population [8]. AF imposes high costs in Europe, e.g., more than one billion EUR in Germany [9, 10], and it accounts for 1% of the United Kingdom’s National Health Service budget [11].

HF is one of the most frequent reasons for hospitalization in Germany. In Mecklenburg-West Pomerania more than 10,000 HF patients annually require inpatient treatment. Lifetime prevalence for HF is 4.2% in the general population. Overall, the diagnosis of HF led to a cost of 2.9 billion EUR in 2006 to the German public health system [12].

Approximately 6.1 million Americans have TRH [13]. TRH is associated with an estimated US$11.3–17.9 billion per year in direct medical expenditures above and beyond expenditures for non-resistant hypertension in the USA.

### Complex interventions in the target diseases atrial fibrillation, heart failure and therapy-resistant hypertension

In patients with HF, complex interventions may lead to a reduction in the re-hospitalization rate by 20–30% [14]. Several studies have investigated the effect of the coordination of medical care between patients, care providers, supporting networks as well as patient training and patient support. Reinforcement of patient self-care and follow-up care in a multidisciplinary team plus telephone support is more effective than telemedicine alone [15–17]. Self-care or self-management includes components, such as diet management, body weight management and early intervention in case of signs of cardiac decompensation [18]. Collaborative work between general practitioners and cardiologists leads to a reduction in mortality of HF patients when compared with the general practitioner being the only physician [19, 20].

Continuous remote monitoring of patients with AF may lead to a reduction of the stroke risk and hospitalization rates for atrial arrhythmias and related stroke [21, 22].

One of the major determinants of poor blood pressure (BP) control results from therapeutic inertia of the physician and suboptimal compliance of the patients. Prevalence estimates of non-adherence in TRH were highly variable (7–66%) [23], and in a recent trial only 20% of the TRH patients took all the medicine that they were prescribed [24]. Meta-analyses of randomized clinical trials (RCTs) demonstrated the efficacy of telemonitoring for BP control [25, 26]. The telemonitoring group had a 31% better target-BP achievement and a significant increase in the use of antihypertensive medications. However, to have a significant BP-lowering effect, the telemonitoring needed to be combined with some sort of intervention, e.g., with active intervention by a health care professional.

### Summary of the evidence

The combination of telemedicine and the reinforcement of patient self-care in a multidisciplinary team together with telephone support are expected to be beneficial in CVDs. This combination of interventions may increase compliance and may allow for early interventions before a serious event has occurred, such as cardiac decompensation in HF patients. This bundle of interventions should be combined with a close collaboration of all treating physicians, and the interaction between general practitioners, resident cardiologists and hospital cardiologists.

### The need for a trial

The novel integrated care concept (NICC) that will be investigated in this trial consists of a bundle of interventions and a specific path for patients of each disease. Boland et al. [27] showed for chronic obstructive pulmonary disease that a pragmatic and non-experimental implementation of a set of interventions resulted in a low level and a wide variety of implementations across different teams. Important barriers to implementation were insufficient motivation of patients, high starting level of care, few patients per disease management team, mild disease levels in the studied population, practicalities of the disease management program and hurdles to reimbursement. Teams also reported unclear instructions, lack of time and lack of motivation. A higher level of implementation was positively associated with improved self-management capabilities, but this association was not found for other outcomes. Other pragmatic studies also reported heterogeneity in the implementation of various interventions [28, 29]. On average, less than 42% of the interventions were implemented, and no team implemented all interventions. However, if only a few interventions are implemented, improvements in patient outcomes cannot be guaranteed [27]. Therefore, as argued by Pinnock et al. [30], after proven efficacy the translation of interventions into a practical service should be evaluated.

### Study objectives and hypotheses

As a result, the superiority of NICC over standard care needs to be shown in a randomized controlled trial (RCT), and we hypothesize that NICC is superior over standard care. The positive effect on survival and/or health in patients with cardiovascular disease is to be demonstrated as a team-based concept for wide acceptance of NICC in practice, which, in turn, would lead to a roll-out of the concept in the federal state of Mecklenburg-West Pomerania and even to all other federal states in Germany.

Our first hypothesis is that NICC is superior to standard care in terms of the clinically relevant events mortality, stroke and myocardial infarction. The event rate for the combined endpoint mortality, stroke or myocardial infarction should thus be lower in the NICC group compared to standard care. The second hypothesis is that NICC leads to a lower number of hospitalizations when compared with standard care. The third hypothesis is that NICC has a lower rate of patients with mortality, stroke, myocardial infarction or cardiac decompensation compared to standard care.

If hospitalizations are reduced through the NICC, we expect that the cost of the NICC will be lower than the cost of standard care. We will, therefore, compare the cost of the NICC with the cost of the standard care.

Another objective of the trial is to show that all components of NICC are implemented in practice for all participating centers.

## Methods

### Study design

CardioCare MV is a prospective, randomized, controlled, parallel-group, open-label with blinded observer, bi-center trial with two groups for comparing NICC with standard care. The study protocol follows the Standard Protocol Items: Recommendations for Interventional Trials (SPIRIT) Statement [31] and reporting will follow the Consolidated Standards of Reporting Trials (CONSORT) Statement [32] and its extension to abstracts [33].

**Fig. 1:**
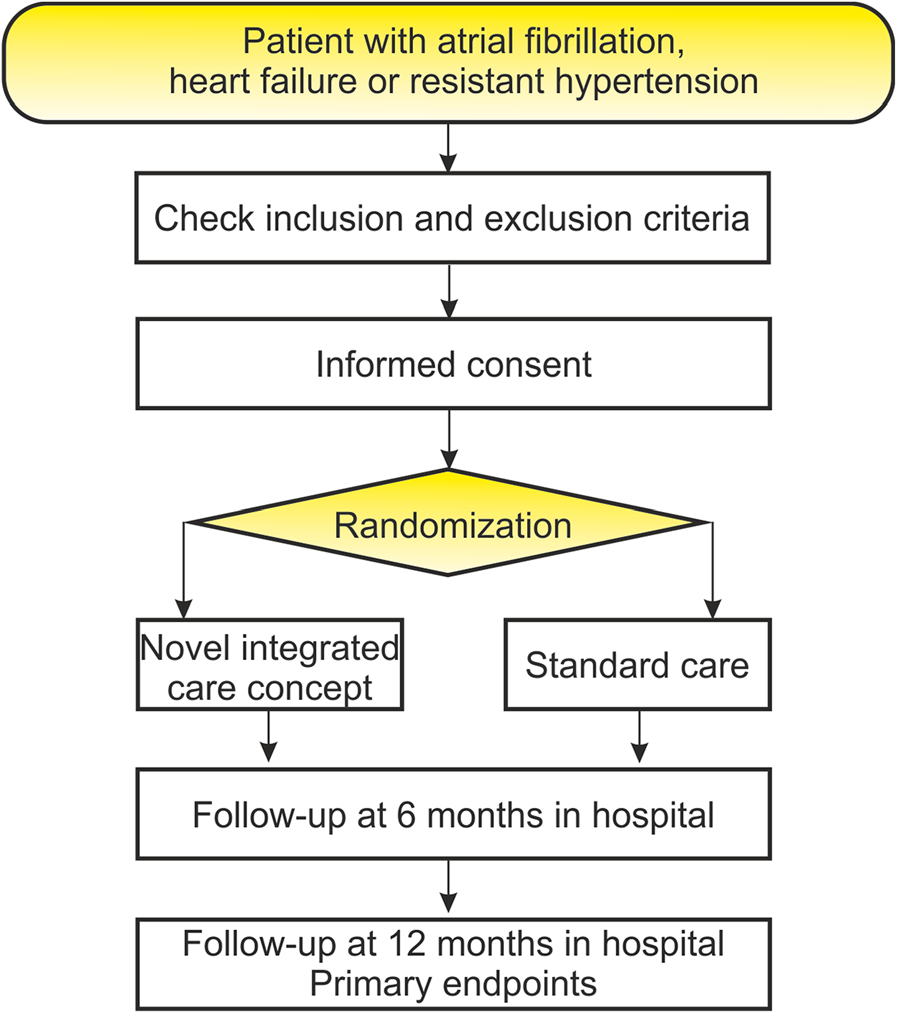
Study flow

Following the SPIRIT Statement we have created Fig. 1 to show the proposed participant flow through the study. The SPIRIT Checklist is shown in Additional file 1.

The study was approved by the Ethics Committee of the Medical Faculty of the University of Rostock (A2017–0117), and it was registered with drks.de on 5 October 2017 (registration number: DRKS00013124). Secondary registration with ClinicalTrials.gov was on 17 October 2017 (registration number: NCT03317951).

### Setting, recruitment, inclusion and exclusion criteria

Patients will be referred by a cardiologist or a general physician to one of the two recruiting centers: University Medical Center Rostock (UMR) or Helios Klinik Schwerin (Schwerin). In addition, inpatients of the UMR or Helios Klinik Schwerin will be included. Trial participants will then be recruited in the two study centers by a cardiologist after diagnosis of AF, HF or TRH. Both centers have long-standing experience in conducting clinical trials and treating patients with CVDs.

Patients will not be remunerated. Referring physicians and referring institutions will receive up to 80 EUR per patient as expense allowance.

Inclusion and exclusion criteria are displayed in Table 1.

**Table 1:** Inclusion and exclusion criteria

Inclusion criteria:
• Heart failure (ICD code I50, NYHA II–IV) or atrial fibrillation (I48, EHRA II–IV) or therapy-resistant hypertension (I10–15 mmHg, ≥ 3 antihypertensives from different drug classes, SBP > 140/90 mmHg or ≥ 4 antihypertensives irrespective of the blood pressure, with at least one drug being a diuretic) • Member of health insurance company Allgemeine Ortskrankenkasse (AOK) Nordost or Techniker Krankenkasse (TK). This is required because patients of the NICC group need to sign an integrated care contract with their health insurance company to allow for legitimate roll-out of intersectoral care delivery model NICC according to German social law • Residence in Mecklenburg-Vorpommern • Age ≥ 18 years • Written informed consent
Exclusion criteria:
• Pregnancy, suspected pregnancy, or breast-feeding period • Participation in another clinical trial up to 30 days before inclusion in this trial • Cognitive deficits: patients need to be able to read and understand the German language as presented on a tablet • Chronic kidney disease requiring dialysis or creatinine clearance < 15 ml/min

*ICD* International Classification of Diseases, *EHRA* European Heart Rhythm Association, *NYHA* New York Heart Association*, NICC* novel integrated care concept, *SBP* systolic blood pressure

### Intervention: the novel integrated care concept (NICC)

**Fig. 2:**
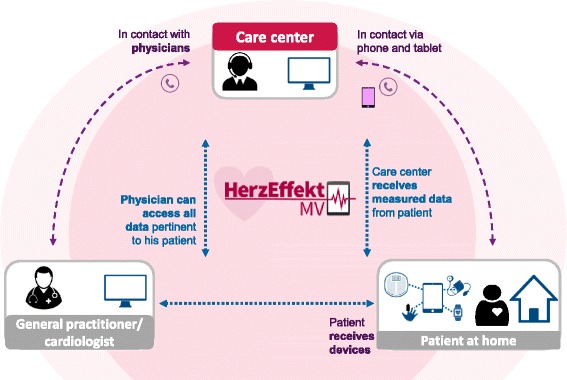
Integration of patients into the novel integrated care concept (NICC) platform

The care center is at the heart of the NICC structure. It will be available round the clock. It is the core platform to share information for all NICC patients in the care process and serves as integration point between the professional groups, as it is expected that all patients of the NICC group will contact first the care center with all questions and needs. The care center is utilizing the NICC platform (Fig. 2) for its care coordination and monitoring of the patient. The NICC platform enables patient management from the distance and allows treating physicians to observe and follow the health status of patients daily.

Using the NICC tablet, patients can provide information from home about their health status via vital-sign measurements and by answering questionnaires using a secure communication channel. Patients will receive feedback about their therapy and their measurements, education about their symptoms and their diseases and reminders and motivation to follow care plans. This allows for a regular evaluation of the patient’s situation – reports are generated at least daily – and risks and a regular review of the therapy and coordination of necessary adjustments with the care providers of the patient.

**Fig. 3:**
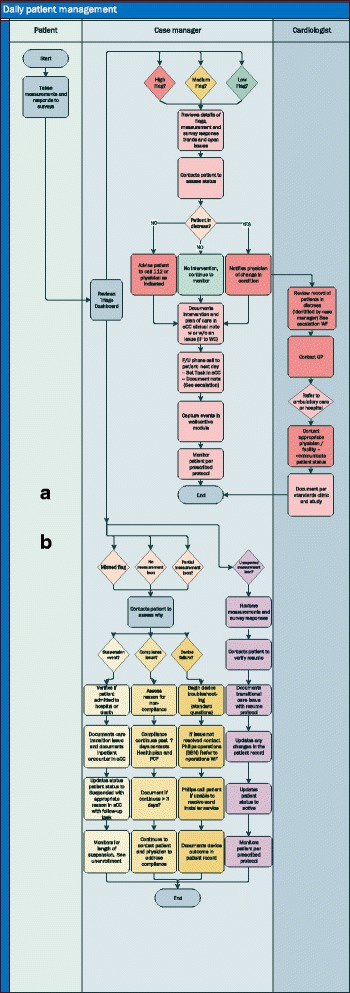
Patient management process

Figure 3 illustrates the patient management process within the study. Patients send their daily health status via vital-sign measurements using a secure communication channel (Fig. 3, upper part). The incoming values will be analyzed automatically by a triage dashboard that reverts to previously set up intervention rules. The results are presented in the form of different flags. Each flag implies a different need of action. For example, a missed measurement can lead to telephone contact with the patient as defined in the workflow. Three different flags (red – high risk, orange – medium risk, yellow – low risk) reflect the urgency of the health status. All flags will be evaluated by the care center medical staff for the need of potential interventions. Values in the normal range or close to normal range will not produce flags.

Flags represent values out of the normal range or critical survey results. The case manager thus has a low to medium to high need for action leading to a process of evaluating the health status of the patient thoroughly. Depending on the risk level of the patient, a clarifying and informing call by the care center medical staff will be performed or other elements of the intervention such as consulting the patient’s treating physician. In some cases, the result may be a physician’s visit or, in very urgent cases, usage of an emergency service. In either case changes in health status will be reported to the treating physician/general practitioner of the patient in written form (Fig. 3, lower part – care center cardiologist).

The general intervention rules are based on the current European Society of Cardiology (ESC) guidelines for treating AF, HF and arterial hypertension, the latter with a special focus on TRH patients.

Communication between the care center and the patient is enabled through a secured line and accessed by the tablet. The patient’s tablet is secured by a user-specific password. From a patient perspective, communicating by tablet will comprise sending measurements and survey results. From the care center’s perspective, communication will include sending and scheduling of surveys to the patient. A case manager calls the patient within a defined interval according to risk-level as the patient’s data or survey responses trigger an outbound activity. The patient can contact the care center by telephone within business hours as required.

Communication between the primary care physician, medical specialists, such as cardiologists, and the care center is defined by workflows, care pathways and care coordination needs. The main communication tools between the different parties are telephone calls, faxes and secure messaging within the software solution, according to the preferences of the involved care giver. Rules and agreements are in place to define when to escalate a patient need to a specialist or inform the patient’s general practitioner.

### Control: standard care

Patients not randomized to NICC will be treated according to current practice as described in the guidelines of the European Society of Cardiology (ESC). For AF, this has been provided by Kirchhof et al. [34]. HF treatment will follow the 2016 ESC guideline for HF [35], and TRH will be treated according to the ESC treatment guideline for arterial hypertension [36].

### Baseline and follow-up examinations

An overview of scheduled study visits is shown in Table 2. Patients will be followed up as outpatients at 6 months and 12 months after randomization. Data will be collected by a physician and a study nurse at the study center who should both be blind to the randomization status. Treatment with NICC will end 12 months after randomization. Long-term follow-up of all study patients is intended at 5 years after randomization.

**Table 2:** Schedule of assessments

	Baseline	Visit at 6 months	Visit at 12 months
Inclusion and exclusion criteria	X		
Informed consent	X		
Randomization	X		
Medical history	X		
Cardiovascular diagnoses	X		
Previous cardiovascular interventions	X		
Comorbidities/cardiovascular risk factors	X	X	X
Physical examination	X	X	X
Laboratory	X	X	X
Resting electrocardiogram	X	X	X
Echocardiography	X	X	X
Medication	X	X	X
Quality of life: EQ-5D-5 L	X	X	X
Heart-related quality of life: HeartQoL	X	X	X
Social support: SSUK-8; Illness-specific Social Support Scale Short version-8	X	X	X
Depression: PHQ-9 (Patient Health Questionnaire depression module)	X	X	X
Anxiety: GAD-7 (Generalized Anxiety Disorder scale)	X	X	X
Well-being: WHO-5 (World Health Organization Well-Being Index)	X	X	X
Medication adherence: MARS-D (Medication Adherence Report Scale) with 5 items	X	X	X
Beliefs in medicines: BMQ (Beliefs about Medicine Questionnaire)	X	X	X
Patient activation: PAM13-D (Patient Activation Measure)	X	X	X
Serious adverse event		X	X
End of study			X

### Primary endpoints

Three primary endpoints will be used in this trial. The first primary endpoint will be the composite endpoint of mortality, stroke and myocardial infarction. The second primary endpoint will be the number of days spent in hospital during the study period. The third primary endpoint will be the composite endpoint of mortality, stroke, myocardial infarction and cardiac decompensation.

### Secondary endpoints

Secondary endpoints include costs, such as stationary medical costs, ambulant medical costs and quality of life as measured with the EQ-5D-5 L [37, 38] and the HeartQoL [39]. Depression (PHQ-9; [40, 41]), anxiety (GAD-7; [42]), well-being (WHO-5; [43]), illness-specific social support (SSUK-8; [44]) and patient activation (PAM13-D; [45]) are among the other secondary endpoints. Safety will be assessed by focusing on serious adverse events (SAEs).

### Randomization

Randomization will be executed in a 1:1 ratio to NICC or standard care using stratified permuted block randomization (PBR) with variable block length. Stratification variables will be diagnosis (AF, HF, TRH) and center (inpatients/outpatients at UMR/Schwerin). After inclusion and exclusion criteria have been checked, the presence of the informed consent form has been ticked and the form has been electronically signed by the physician, the randomization result will be displayed in the trial database.

### Statistical analysis

All statistical analyses will be described in detail in a statistical analysis plan (SAP) which will be finalized before the randomization of the last patient. Analysis populations for the primary endpoints will be the full analysis set (FAS) based on the intention-to-treat (ITT) principle. Neither interim analyses nor adaptations are planned for this trial.

The familywise error rate is set to 5%. All tests will be two-sided. The following multiple testing procedure will be used for the three primary endpoints. The first and second endpoint will be tested at the 2.5% test level. If any of the first two primary endpoints is significant at the two-sided 2.5% test level, the other hypothesis for the first two primary endpoints will be tested at the 5% test level. If both the first and second primary endpoints are significant, the third primary endpoint will be tested at the 5% test level. No other adjustments will be made for multiple testing.

The first and the third primary endpoints will be analyzed using logistic regression with adjustment for the stratification variables. A Poisson regression allowing for over- or underdispersion with adjustment for stratification variables will be estimated for the second primary endpoint. Two-sided asymptotic Wald tests and corresponding confidence intervals will be calculated. All secondary endpoints will be tested by appropriate tests and models exploratorily using the two-sided 5% significance level.

Missing values for the primary endpoints will be minimized by intensive follow-up, such as telephone calls by the call center. Missing values for the primary endpoints will be imputed using MICE [46]. Missing data of scores from questionnaires will be handled according to the respective manual.

### Sample size calculations

The aim of the trial is to demonstrate superiority of NICC when compared with standard care. For sample size calculations for the first primary endpoint, we assumed an event rate of 0.08 in the standard care group [12]. For comparing two binomially distributed proportions using the Yates correction [47], the required sample size is approximately 1465 per group in case of approximately 8.5% dropouts, 1:1 individual allocation ratio, a two-sided 2.5% test level and 80% power for demonstrating an absolute reduction of the event rate by 3% for the NICC group. The total required sample size thus is approximately 2930 patients.

The second primary endpoint will be tested for superiority at the two-sided 2.5% test level. The number of HF hospitalizations in Germany was approximately 400,000 in 2013 [48]. The age- and sex-standardized HF prevalence in Germany is approximately 1.7% [49] so that approximately 1,360,000 Germany suffer from HF, yielding a hospitalization intensity of approximately 400,000/1,360,000, i.e., about 0.3. Clinically relevant would be a reduction by one third to 0.2. The expected number of hospitalizations per group are 405 for the control group and 270 for the NICC group with a total sample size of 2930 including dropouts, resulting in power to detect a difference in the number of hospitalizations exceeding 99.8% at the two-sided 2.5% test level. If the sample size of 2930 patients cannot be reached for any reason, the power to detect a difference in hospitalization rates of 0.3 and 0.2 would be 80% with the other parameters as before if at least 150 hospitalizations occur in the control group and 100 hospitalizations in the NICC group. The required sample size for these frequencies would be approximately 543 subjects per group, yielding a total required sample size of approximately 1086 patients.

### Data management and data monitoring

Data security aspects are described in detail in a separate data protection concept. Three different types of data will be collected for this trial, (1) data collected in the electronic Case Report Form (eCRF), (2) data from the participating health insurance companies and (3) data from the NICC center in Rostock. In addition, for patients in the intervention group data will be collected on the Philips devices.

The trial database and the eCRF will be developed, maintained and hosted by AMEDON GmbH using standard operating procedures (SOPs) from AMEDON GmbH. Patient data will be stored only pseudonymized in the AMEDON database.

Only pseudonymized routine data from health insurance companies will be integrated into the trial database using a trustee concept.

The NICC platform and databases will be developed, maintained and hosted by Philips GmbH and its suppliers. The platform and databases are integrated into a general Philips product security and safety concept. Personal and anamnestic patient data are treated as confidential and will be stored fully encrypted in the Philips NICC databases. Data stored on the patient tablet provided by Philips GmbH are encrypted with the encryption key bound to the patients’ credentials. Patient tablets are locked-down so that patients can neither change the configuration nor install additional apps. Tablet data will be integrated into the trial database by a physician working in the care center using pseudonymized data.

All relevant study data will be archived by the sponsor of the study for at least 10 years after end of study.

AMEDON GmbH will conduct clinical onsite monitoring to ensure patients’ rights, patients’ security and reliability of trial results. Informed consent and defined key data will be checked for approximately 10% patients. The medical file will be screened for serious adverse events for approximately 3% of the patients.

In presentations or publications arising from this study, information will be provided in such a way that individual participants cannot be identified. The local centers are entitled to use the recorded data for additional scientific exploitation under their own name, but not before the main results have been published. There are no exceptions to this rule. Any sub-publication requires approval by the sponsor.

### Governance

The whole project is supervised by a Steering Committee. The Steering Committee met almost monthly in 2017.

An independent Data Monitoring Committee (DMC), consisting of two cardiologists and one biostatistician, will be established. The task of the DMC is to oversee the safety of the trial subjects in the clinical trial by assessing the safety and efficacy of the care concepts, and to monitor the integrity and validity of the data collected and the conduct of the clinical trial.

## Discussion

Morbidity and mortality rates from CVDs are high in Mecklenburg-West Pomerania compared to less rural federal states in Germany. Physicians and hospitals are more difficult to reach compared to urban areas. The combination of telemedicine and the reinforcement of patient self-care in a multidisciplinary team together with telephone support may reduce morbidity and mortality.

We have, therefore, developed a novel care concept with its core being a care center. The care center has two main functions. It will serve as integration point between the professional groups, and it is intended to be the first contact point for patients in case of questions and needs. With the help of the care center which will be available round the clock we aim to increase the treatment compliance. Even more, it may allow for early interventions before a serious event occurs. We expect that the cost of the NICC will be lower than standard care because of reduced hospitalizations.

A risk of the trial is its short treatment and observation period of 1 year per patient within the trial. Effects on mortality may not be identifiable in this short term. However, an expansion was not possible because of the funding line which requires a completion of the project within the year 2019. We therefore aim at performing a follow-up at 5 years after last patient inclusion.

CardioCare MV will allow for evidence-based political and economic decision-making. If we can demonstrate positive effects of the NICC, NICC will be immediately rolled out to the entire state of Mecklenburg-West Pomerania and become standard of care. The trial will also guide additional research to disentangle the effects of this complex intervention. In the near future, we would like to expand NICC to other CVDs such as stroke, and other indications such as chronic pain.

### Trial status

The trial opened for accrual on 1 December 2017. First patient included was on 4 December 2017. Accrual is planned to be completed by the end of December 2018.

## Additional file

Additional file 1: SPIRIT 2013 Checklist applied to CardioCare MV study protocol. (DOC 186 kb)

## Abbreviations

AF: Atrial fibrillation
CVD: Cardiovascular disease
ESC: European Society of Cardiology
HF: Heart failure
NICC: Novel integrated care concept
RCT: Randomized controlled trial
TRH: Treatment-resistant hypertension
UMR: University Medical Center Rostock

## References

[1] Cardiovascular diseases (CVDs): Fact Sheet. Updated May 2017. http://www.who.int/mediacentre/factsheets/fs317/en/. Accessed 18 Feb 2018.

[2] Tarride JE, Lim M, DesMeules M, Luo W, Burke N, O’Reilly D, Bowen J, Goeree R (2009). A review of the cost of cardiovascular disease. Can J Cardiol.

[3] Gesundheit in Deutschland. Gesundheitsberichterstattung des Bundes. Gemeinsam getragen von RKI und Destatis. https://www.rki.de/DE/Content/Gesundheitsmonitoring/Gesundheitsberichterstattung/GesInDtld/gesundheit_in_deutschland_2015.html. Accessed 18 Feb 2018.

[4] Miller NH, Hill M, Kottke T, Ockene IS (1997). The multilevel compliance challenge: recommendations for a call to action. A statement for healthcare professionals. Circulation.

[5] Werz N, Andersen U, Woyke W (2013). Land Mecklenburg-Vorpommern. Handwörterbuch des politischen Systems der Bundesrepublik Deutschland.

[6] Meinertz T, Diegeler A, Stiller B, Fleck E, Heinemann MK, Schmaltz AA, Vestweber M, Bestehorn K, Beckmann A, Hamm C, Cremer J (2015). German Heart Report 2013. Clin Res Cardiol.

[7] Chugh SS, Havmoeller R, Narayanan K, Singh D, Rienstra M, Benjamin EJ, Gillum RF, Kim YH, McAnulty JH, Zheng ZJ (2014). Worldwide epidemiology of atrial fibrillation: a Global Burden of Disease 2010 Study. Circulation.

[8] Schuchert A, Gerth A, Näbauer M, Steinbeck G, Meinertz T (2005). Vorhofflimmern: Epidemiologie, Klinik und Prognose [Atrial fibrillation: epidemiology, clinic and prognosis]. Med Welt.

[9] McBride D, Mattenklotz AM, Willich SN, Brüggenjürgen B (2009). The costs of care in atrial fibrillation and the effect of treatment modalities in Germany. Value Health.

[10] Taylor MJ, Scuffham PA, McCollam PL, Newby DE (2007). Acute coronary syndromes in Europe: 1-year costs and outcomes. Curr Med Res Opin.

[11] Stewart S, Murphy NF, Walker A, McGuire A, McMurray JJ (2004). Cost of an emerging epidemic: an economic analysis of atrial fibrillation in the UK. Heart.

[12] Neumann T, Biermann J, Erbel R, Neumann A, Wasem J, Ertl G, Dietz R (2009). Heart failure: the commonest reason for hospital admission in Germany: medical and economic perspectives. Dtsch Arztebl Int.

[13] Ahmed MI, Calhoun DA (2011). Resistant hypertension: bad and getting worse. Hypertension.

[14] McKelvie RS. Heart failure. BMJ Clin Evid. 2010;2010 PMC2907608 21718583

[15] McAlister FA, Stewart S, Ferrua S, McMurray JJ (2004). Multidisciplinary strategies for the management of heart failure patients at high risk for admission: a systematic review of randomized trials. J Am Coll Cardiol.

[16] Holland R, Battersby J, Harvey I, Lenaghan E, Smith J, Hay L (2005). Systematic review of multidisciplinary interventions in heart failure. Heart.

[17] Wennberg DE, Marr A, Lang L, O’Malley S, Bennett G (2010). A randomized trial of a telephone care-management strategy. N Engl J Med.

[18] Jovicic A, Holroyd-Leduc JM, Straus SE (2006). Effects of self-management intervention on health outcomes of patients with heart failure: a systematic review of randomized controlled trials. BMC Cardiovasc Disord.

[19] Lee DS, Stukel TA, Austin PC, Alter DA, Schull MJ, You JJ, Chong A, Henry D, Tu JV (2010). Improved outcomes with early collaborative care of ambulatory heart failure patients discharged from the emergency department. Circulation.

[20] Indridason OS, Coffman CJ, Oddone EZ (2003). Is specialty care associated with improved survival of patients with congestive heart failure?. Am Heart J.

[21] Ricci RP (2013). Disease management: atrial fibrillation and home monitoring. Europace.

[22] Mabo P, Victor F, Bazin P, Ahres S, Babuty D, Da Costa A, Binet D, Daubert JC, Compas Trial Investigators (2012). A randomized trial of long-term remote monitoring of pacemaker recipients (the COMPAS trial). Eur Heart J.

[23] Hyman DJ, Pavlik V (2015). Medication adherence and resistant hypertension. J Hum Hypertens.

[24] de Jager RL, de Beus E, Beeftink MM, Sanders MF, Vonken EJ, Voskuil M, van Maarseveen EM, Bots ML, Blankestijn PJ (2017). Investigators Study. Impact of medication adherence on the effect of renal denervation: the SYMPATHY trial. Hypertension.

[25] Omboni S, Guarda A (2011). Impact of home blood pressure telemonitoring and blood pressure control: a meta-analysis of randomized controlled studies. Am J Hypertens.

[26] Omboni S, Gazzola T, Carabelli G, Parati G (2013). Clinical usefulness and cost effectiveness of home blood pressure telemonitoring: meta-analysis of randomized controlled studies. J Hypertens.

[27] Boland MR, Kruis AL, Huygens SA, Tsiachristas A, Assendelft WJ, Gussekloo J, Blom CM, Chavannes NH, Rutten-van Molken MP (2015). Exploring the variation in implementation of a COPD disease management programme and its impact on health outcomes: a post hoc analysis of the RECODE cluster randomised trial. NPJ Prim Care Respir Med.

[28] Bodenheimer T, Wagner EH, Grumbach K (2002). Improving primary care for patients with chronic illness: the chronic care model, Part 2. JAMA.

[29] Pearson ML, Wu S, Schaefer J, Bonomi AE, Shortell SM, Mendel PJ, Marsteller JA, Louis TA, Rosen M, Keeler EB (2005). Assessing the implementation of the chronic care model in quality improvement collaboratives. Health Serv Res.

[30] Pinnock H, Epiphaniou E, Taylor SJ, Phase IV – implementation studies (2014). The forgotten finale to the complex intervention methodology framework. Ann Am Thorac Soc.

[31] Chan AW, Tetzlaff JM, Altman DG, Laupacis A, Gøtzsche PC, Krleža-Jerić K, Hróbjartsson AA, Mann H, Dickersin K, Berlin JA (2013). SPIRIT 2013 statement: defining standard protocol items for clinical trials. Ann Intern Med.

[32] Schulz KF, Altman DG, Moher D, for the CONSORT Group (2010). CONSORT 2010 Statement: Updated Guidelines for Reporting Parallel Group Randomised Trials. PLoS Med.

[33] Hopewell S, Clarke M, Moher D, Wager E, Middleton P, Altman DG, Schulz KF (2008). CONSORT for reporting randomised trials in journal and conference abstracts. Lancet.

[34] Kirchhof P, Benussi S, Kotecha D, Ahlsson A, Atar D, Casadei B, Castella M, Diener HC, Heidbuchel H, Hendriks J (2016). ESC Guidelines for the management of atrial fibrillation developed in collaboration with EACTS. Eur Heart J.

[35] Ponikowski P, Voors AA, Anker SD, Bueno H, Cleland JG, Coats AJ, Falk V, Gonzalez-Juanatey JR, Harjola VP, Jankowska EA (2016). ESC Guidelines for the diagnosis and treatment of acute and chronic heart failure: The Task Force for the diagnosis and treatment of acute and chronic heart failure of the European Society of Cardiology (ESC) – Developed with the special contribution of the Heart Failure Association (HFA) of the ESC. Eur Heart J.

[36] Mancia G, Fagard R, Narkiewicz K, Redon J, Zanchetti A, Bohm M, Christiaens T, Cifkova R, De Backer G, Dominiczak A (2013). ESH/ESC guidelines for the management of arterial hypertension: the Task Force for the Management of Arterial Hypertension of the European Society of Hypertension (ESH) and of the European Society of Cardiology (ESC). Eur Heart J.

[37] Brooks R (1996). EuroQol: the current state of play. Health Policy.

[38] Devlin NJ, Brooks R (2017). EQ-5D and the EuroQol Group: past, present and future. Appl Health Econ Health Policy.

[39] Oldridge N, Höfer S, McGee H, Conroy R, Doyle F, Saner H (2014). The HeartQoL: part II. Validation of a new core health-related quality of life questionnaire for patients with ischemic heart disease. Eur J Prev Cardiol.

[40] Löwe B, Spitzer RL, Zipfel S, Herzog W (2002). Gesundheitsfragebogen für Patienten (PHQ-D). Komplettversion und Kurzform. Testmappe mit Manual, Fragebögen, Schablonen.

[41] Gräfe K, Zipfel S, Herzog W, Löwe B (2004). Screening psychischer Störungen mit dem “Gesundheitsfragebogen für Patienten (PHQ-D)”. Diagnostica.

[42] Löwe B, Decker O, Müller S, Brahler E, Schellberg D, Herzog W, Herzberg PY (2008). Validation and standardization of the Generalized Anxiety Disorder Screener (GAD-7) in the general population. Med Care.

[43] Topp CW, Østergaard SD, Søndergaard S, Bech P (2015). The WHO-5 Well-Being Index: a systematic review of the literature. Psychother Psychosom.

[44] Ullrich A, Mehnert A (2010). Psychometrische Evaluation and Validierung einer 8-Item-Kurzversion der Skalen zur Sozialen Unterstützung bei Krankheit (SSUK) bei Krebspatienten. Klin Diagn Eval.

[45] Zill JM, Dwinger S, Kriston L, Rohenkohl A, Harter M, Dirmaier J (2013). Psychometric evaluation of the German version of the Patient Activation Measure (PAM13). BMC Public Health.

[46] Azur MJ, Stuart EA, Frangakis C, Leaf PJ (2011). Multiple imputation by chained equations: what is it and how does it work?. Int J Methods Psychiatr Res.

[47] Bristol DR (1989). Sample sizes for constructing confidence intervals and testing hypotheses. Stat Med.

[48] Christ M, Störk S, Dörr M, Heppner HJ, Müller C, Wachter R, Riemer U, Trend HF (2016). Germany Project. Heart failure epidemiology 2000–2013: insights from the German Federal Health Monitoring System. Eur J Heart Fail.

[49] Ohlmeier C, Mikolajczyk R, Frick J, Prütz F, Haverkamp W, Garbe E (2015). Incidence, prevalence and 1-year all-cause mortality of heart failure in Germany: a study based on electronic healthcare data of more than six million persons. Clin Res Cardiol.

