# Supplementary material for: A novel integrated care concept (NICC) versus standard care in the treatment of chronic cardiovascular diseases: protocol for the randomized controlled trial CardioCare MV
Source: Trials. 2018 Feb 20;19:120. doi: 10.1186/s13063-018-2502-1 (PMC5819165; doi:10.1186/s13063-018-2502-1)
Supplement: Supplementary file 1 — SPIRIT 2013 Checklist applied to CardioCare MV study protocol. (DOC 186 kb) [file 13063_2018_2502_MOESM1_ESM.doc]

Additional file 1 SPIRIT 2013 Checklist applied to CardioCare MV study protocol

| Section/item | Item No | Description | Explanation | Addressed in study protocol, section |
| --- | --- | --- | --- | --- |
| **Administrative information** | | |  |  |
| Title | 1 | Descriptive title identifying the study design, population, interventions, and, if applicable, trial acronym | A novel integrated care concept (NICC) versus standard care in the treatment of chronic cardiovascular diseases: protocol for the randomized controlled trial CardioCare MV | Synopsis, p. 7 |
| Trial registration | 2a | Trial identifier and registry name. If not yet registered, name of intended registry | DRKS00013124, DRKS, Oct 05, 2017 | Synopsis, p. 9 |
|  | 2b | All items from the World Health Organization Trial Registration Data Set | See table at the bottom of this document | Provided as separate table |
| Protocol version | 3 | Date and version identifier | November 13, 2017, version 2.0 | Title page |
| Funding | 4 | Sources and types of financial, material, and other support | This trial will be financed by the Gemeinsamer Bundesausschuss (G-BA) within the project HerzEffekt MV (funding code: 01NVF16003). Standard care and telemedicine devices will be paid by health insurance companies. Cost for medical examinations and laboratory measurements will be shared by the Universitätsmedizin Rostock and health insurance companies. | Section 6 |
| Roles and responsibilities | 5a | Names, affiliations, and roles of protocol contributors | If the trial design will be published in an internationally recognized journal, the following author contributions will be included in the article:  CS, MA, BB, KK, MM, AÖ and AZ jointly conceived the study. AZ developed the study design. AB, GH and HN contributed to the selection of the standard inventories. AÖ, MM, KK and BB developed the electronic case report forms. BB led the development of the data protection concept. All authors contributed to the refinement of the study protocol. The funding agency had no role in the design of the study and the study protocol.  The following order of author names will be used in the publication:  C Schmidt, A Öner, M Mann, K Krockenberger (all four co-first authors),  M Abbondanzieri, B Brandewiede, A Brüge, G Hostenkamp, A Kaiser, H Neumeyer, (authors in alphabetical order)  A Ziegler (senior author).  Corresponding authors will be CS and AZ. | Section 3 |
|  | 5b | Name and contact information for the trial sponsor | Universitätsmedizin Rostock  Ernst-Heydemann-Str. 8  18057 Rostock, Germany  Represented by:  Prof. Dr. med. Christian Schmidt (Ärztlicher Vorstand und Vorstandsvorsitzender)  Phone: +49 381 494 5011  Fax: +49 381 494 5012  E-Mail: christian.schmidt@med.uni-rostock.de | Section 2 |
|  | 5c | Role of study sponsor and funders, if any, in study design; collection, management, analysis, and interpretation of data; writing of the report; and the decision to submit the report for publication, including whether they will have ultimate authority over any of these activities | The funding agency had no role in the design of the study and the study protocol. | Section 3 |
|  | 5d | Composition, roles, and responsibilities of the coordinating centre, steering committee, endpoint adjudication committee, data management team, and other individuals or groups overseeing the trial, if applicable (see Item 21a for data monitoring committee) | Trial management, central data management, regulatory affairs, monitoring, data management: Bernard Brandewiede, AMEDON GmbH, Willy-Brandt-Allee 31c, 23554 Lübeck, Germany  Dr. sc. hum. Katja Krockenberger, AMEDON GmbH, Willy-Brandt-Allee 31c, 23554 Lübeck, Germany  Socioeconomic analyses: Elias Olshausen, Lohfert & Lohfert AG, Rothenbaumchaussee 76, 20148 Hamburg, Germany | Section 2 (composition of DMC), Section 16.1 |
| Introduction |  |  |  |  |
| Background and rationale | 6a | Description of research question and justification for undertaking the trial, including summary of relevant studies (published and unpublished) examining benefits and harms for each intervention | See manuscript, background | Section 7 |
|  | 6b | Explanation for choice of comparators | See manuscript, background, study objectives | Section 7 |
| Objectives | 7 | Specific objectives or hypotheses | See manuscript, background, study objectives | Section 8 |
| Trial design | 8 | Description of trial design including type of trial (eg, parallel group, crossover, factorial, single group), allocation ratio, and framework (eg, superiority, equivalence, noninferiority, exploratory) | CardioCare MV is a prospective, randomized, controlled, parallel-group, open, bicenter trial with two groups for comparing NICC with standard care. | Section 9 |
| Methods: Participants, interventions, and outcomes | | |  |  |
| Study setting | 9 | Description of study settings (eg, community clinic, academic hospital) and list of countries where data will be collected. Reference to where list of study sites can be obtained | Patients will be referred by a cardiologist or a general physician to one of the two recruiting centers University Medical Center Rostock (UMR) or Helios Klinik Schwerin (Schwerin). In addition, inpatients of the UMR or Helios Klinik Schwerin will be included. Trial participants will then be recruited in the two study centers by a cardiologist after diagnosis of AF, HF or TRH. Both centers have long-standing experience in conducting clinical trials and treating patients with CVDs. | Section 10 |
| Eligibility criteria | 10 | Inclusion and exclusion criteria for participants. If applicable, eligibility criteria for study centres and individuals who will perform the interventions (eg, surgeons, psychotherapists) | Manuscript, box 1 | Sections 9.1 and 9.2 |
| Interventions | 11a | Interventions for each group with sufficient detail to allow replication, including how and when they will be administered | Manuscript, methods, intervention: the novel integrated care concept (NICC)  and  Manuscript, methods, control: standard care | Sections 11.3 and 11.4 |
|  | 11b | Criteria for discontinuing or modifying allocated interventions for a given trial participant (eg, drug dose change in response to harms, participant request, or improving/worsening disease) | Participation will stop if the patient changes the health insurance company so that an integrated care contract is no longer available. Participation in the study is voluntary, and a patient may refuse to participate or withdraw from the trial, at any time, without penalty or loss of benefits to which the patient is otherwise entitled. | Section 18.6 |
|  | 11c | Strategies to improve adherence to intervention protocols, and any procedures for monitoring adherence (eg, drug tablet return, laboratory tests) | Use of the tablets will be monitored. Patients will be contacted by the care centre in case of no feedback by patient. | Sections 11.3, 12.2.3 and 12.2.12 |
|  | 11d | Relevant concomitant care and interventions that are permitted or prohibited during the trial | Concomitant care is standard care according to ESC guidelines. | Section 11.4 |
| Outcomes | 12 | Primary, secondary, and other outcomes, including the specific measurement variable (eg, systolic blood pressure), analysis metric (eg, change from baseline, final value, time to event), method of aggregation (eg, median, proportion), and time point for each outcome. Explanation of the clinical relevance of chosen efficacy and harm outcomes is strongly recommended | The first primary endpoint will be the composite endpoint of mortality, stroke and myocardial infarction, measured at 12 months after randomization.  The second primary endpoint will be the number of days spent in hospital, measured at 12 months after randomization.  The third primary endpoint will be the composite endpoint of mortality, stroke, myocardial infarction and cardiac decompensation, measured at 12 months after randomization.  Secondary endpoints listed in manuscript, methods, secondary endpoints.  No additional explanation provided for relevance of the three primary endpoints as all are immediately clinically relevant | Sections 12 and 15 |
| Participant timeline | 13 | Time schedule of enrolment, interventions (including any run-ins and washouts), assessments, and visits for participants. A schematic diagram is highly recommended (see Figure) | Supplementary Figure 1 displayed in a separate file | Section 9.1 (Figure 1) and Section 11.1 (Figure 2) |
| Sample size | 14 | Estimated number of participants needed to achieve study objectives and how it was determined, including clinical and statistical assumptions supporting any sample size calculations | Manuscript, methods, sample size calculations | Section 15.3.2 |
| Recruitment | 15 | Strategies for achieving adequate participant enrolment to reach target sample size | Access to patients, e.g., through hospitals, treating physicians and cardiologists, media campaign | Section 10 |
| **Methods: Assignment of interventions (for controlled trials)** | | |  |  |
| Allocation: |  |  |  |  |
| Sequence generation | 16a | Method of generating the allocation sequence (eg, computer-generated random numbers), and list of any factors for stratification. To reduce predictability of a random sequence, details of any planned restriction (eg, blocking) should be provided in a separate document that is unavailable to those who enrol participants or assign interventions | Permute block randomization with variable block length, stratified by centre, mode of admission (treating physician, hospital with primary diagnosis cardiovascular diagnosis, hospital with secondary diagnosis cardiovascular diagnosis) and cardiovascular diagnosis (AF, HF; TRH) | Sections 11.2 and 13 |
| Allocation concealment mechanism | 16b | Mechanism of implementing the allocation sequence (eg, central telephone; sequentially numbered, opaque, sealed envelopes), describing any steps to conceal the sequence until interventions are assigned | Randomization electronically in eCRF database. Randomization only after formal ticking of inclusion/exclusion criteria, including informed consent and IV contract of health insurance | Sections 11.2 and 13 |
| Implementation | 16c | Who will generate the allocation sequence, who will enrol participants, and who will assign participants to interventions | Generation of randomization list: biostatistician; transfer of randomization lists into trial database: data manager; participant enrolment: cardiologist; assignment: electronically; result displayed to cardiologist | Section 13 |
| Blinding (masking) | 17a | Who will be blinded after assignment to interventions (eg, trial participants, care providers, outcome assessors, data analysts), and how | Open label | Not applicable |
|  | 17b | If blinded, circumstances under which unblinding is permissible, and procedure for revealing a participant’s allocated intervention during the trial | N.A. | Not applicable |
| **Methods: Data collection, management, and analysis** | | |  |  |
| Data collection methods | 18a | Plans for assessment and collection of outcome, baseline, and other trial data, including any related processes to promote data quality (eg, duplicate measurements, training of assessors) and a description of study instruments (eg, questionnaires, laboratory tests) along with their reliability and validity, if known. Reference to where data collection forms can be found, if not in the protocol | Manuscript, table 1 | Section 17 |
|  | 18b | Plans to promote participant retention and complete follow-up, including list of any outcome data to be collected for participants who discontinue or deviate from intervention protocols | Even if participants may no longer participate in the trial (e.g., move out of Mecklenburg West Pomerania), it is intended to follow-up all patients at scheduled follow-ups.  Participants will be reminded of scheduled follow-up approx. 1 month before the scheduled date | Sections 15.1 and 8.3 |
| Data management | 19 | Plans for data entry, coding, security, and storage, including any related processes to promote data quality (eg, double data entry; range checks for data values). Reference to where details of data management procedures can be found, if not in the protocol | eCRF database | Section 17 |
| Statistical methods | 20a | Statistical methods for analysing primary and secondary outcomes. Reference to where other details of the statistical analysis plan can be found, if not in the protocol | Statistical analysis plan will be finalized prior to randomization of the last patient.  Manuscript, methods, statistical analysis. | Section 15 |
|  | 20b | Methods for any additional analyses (eg, subgroup and adjusted analyses) | Manuscript, methods, statistical analysis | 15.3.3 |
|  | 20c | Definition of analysis population relating to protocol non-adherence (eg, as randomised analysis), and any statistical methods to handle missing data (eg, multiple imputation) | Manuscript, methods, statistical analysis | Section 15.1 |
| **Methods: Monitoring** | | |  |  |
| Data monitoring | 21a | Composition of data monitoring committee (DMC); summary of its role and reporting structure; statement of whether it is independent from the sponsor and competing interests; and reference to where further details about its charter can be found, if not in the protocol. Alternatively, an explanation of why a DMC is not needed | Charter not finalized yet.  Independence from sponsor established by selecting members not belonging to the institution of the sponsor.  Two cardiologists and one biostatistician form the DMC. | Sections 2 and 16.1 |
|  | 21b | Description of any interim analyses and stopping guidelines, including who will have access to these interim results and make the final decision to terminate the trial | Neither interim analyses nor adaptations planned. | Section 15.3.1 |
| Harms | 22 | Plans for collecting, assessing, reporting, and managing solicited and spontaneously reported adverse events and other unintended effects of trial interventions or trial conduct | Only SAEs will be recorded because the tablets and devices for measuring blood pressure etc. are considered to be safe. | Sections 14, 15.5, 18.8 |
| Auditing | 23 | Frequency and procedures for auditing trial conduct, if any, and whether the process will be independent from investigators and the sponsor | No audits planned. | Section 17.3 |
| Ethics and dissemination | | |  |  |
| Research ethics approval | 24 | Plans for seeking research ethics committee/institutional review board (REC/IRB) approval | Approval obtained. Ethics approval was obtained from the ethics committee of the Medical Faculty of the University of Rostock on July 18, 2017, and its registration number is A 2017-0117. | Section 18.1 |
| Protocol amendments | 25 | Plans for communicating important protocol modifications (eg, changes to eligibility criteria, outcomes, analyses) to relevant parties (eg, investigators, REC/IRBs, trial participants, trial registries, journals, regulators) | Amendments will be submitted to the ethics committee and noted in the trial registry. | Section 18.5 |
| Consent or assent | 26a | Who will obtain informed consent or assent from potential trial participants or authorised surrogates, and how (see Item 32) | Care centre cardiologist | Section 10 |
|  | 26b | Additional consent provisions for collection and use of participant data and biological specimens in ancillary studies, if applicable | N.A. | Sections 17.2 and 17.6 |
| Confidentiality | 27 | How personal information about potential and enrolled participants will be collected, shared, and maintained in order to protect confidentiality before, during, and after the trial | Specific data protection concept developed that has been approved by the data protection officer of the state Mecklenburg West Pomerania | Section 17 |
| Declaration of interests | 28 | Financial and other competing interests for principal investigators for the overall trial and each study site | No competing interests declared. | Section 18.4 |
| Access to data | 29 | Statement of who will have access to the final trial dataset, and disclosure of contractual agreements that limit such access for investigators | Access to final dataset to trial biostatistician. Socioeconomic data stored in data warehouse available to socioeconomic analyst. | Section 17 |
| Ancillary and post-trial care | 30 | Provisions, if any, for ancillary and post-trial care, and for compensation to those who suffer harm from trial participation | N.A. | Not applicable |
| Dissemination policy | 31a | Plans for investigators and sponsor to communicate trial results to participants, healthcare professionals, the public, and other relevant groups (eg, via publication, reporting in results databases, or other data sharing arrangements), including any publication restrictions | The scientific results will be published in international, peer-reviewed journals of the highest possible quality, and they will follow publication statements. In addition, results will be presented at medical congresses and symposia. For methodological reasons, results of the trial will be published only after study database closure. | Section 18.10 |
|  | 31b | Authorship eligibility guidelines and any intended use of professional writers | Manuscript authorship will be selected according to the requirements of the New England Journal of Medicine (http://www.icmje.org/).  The use of a professional writer is not intended. | Section 18.10 |
|  | 31c | Plans, if any, for granting public access to the full protocol, participant-level dataset, and statistical code | Full protocol available upon request from the corresponding authors. Access to participant-level data and statistical analysis code may not be granted. | Not applicable |
| Appendices |  |  |  |  |
| Informed consent materials | 32 | Model consent form and other related documentation given to participants and authorised surrogates | Provided in the study protocol | Section 19 |
| Biological specimens | 33 | Plans for collection, laboratory evaluation, and storage of biological specimens for genetic or molecular analysis in the current trial and for future use in ancillary studies, if applicable | N.A. | Not applicable |

Table. All items from the World Health Organization Trial Registration Data Set

| **Primary Registry and Trial Identifying Number** | drks.de, registration number: DRKS00013124 |
| --- | --- |
| **Date of Registration in Primary Registry** | Oct 05, 2017 |
| **Secondary Identifying Numbers** | Funding code of the Federal Joint Committee (Gemeinsamer Bundesausschuss): 01NVF16003  Registration number of the ethics committee of the medical faculty of University of Rostock: A 2017-0117 |
| **Sources of Monetary or Material Support** | - Federal Joint Committee (Gemeinsamer Bundesausschuss): 01NVF16003. - Standard care and telemedicine devices will be paid by health insurance companies. - Cost for medical examinations and laboratory measurements will be shared by the Universitätsmedizin Rostock and health insurance companies. |
| **Primary Sponsor** | University Medicine Rostock, Ernst-Heydemann-Str. 8, 18057 Rostock, Germany |
| **Secondary Sponsor(s)** | - Coordinating Investigator: Prof. Dr. Christian Schmidt, Ärztlicher Vorstand und Vorstandsvorsitzender, Universitätsmedizin Rostock, Ernst-Heydemann-Str. 8, 18057 Rostock, Germany - Deputy Coordinating Investigator: Priv.-Doz. Dr. Alper Öner, Abteilung Kardiologie, Universitätsmedizin Rostock, Ernst-Heydemann-Str. 8, 18057 Rostock, Germany - Medical Investigator Rostock: Priv.-Doz. Dr. Alper Öner, Abteilung Kardiologie, Universitätsmedizin Rostock, Ernst-Heydemann-Str. 8, 18057 Rostock, Germany - Medical Investigator Schwerin: Prof. Dr. Alexander Staudt, Herzzentrum Nord-Ost, Helios Kliniken Schwerin, Wismarsche Str. 393-397, 19055 Schwerin, Germany - Biostatistician: Prof. Dr. Andreas Ziegler, StatSol, Moenring 2, 23560 Lübeck, Germany - Trial Management, Central Data Management, Regulatory Affairs, Monitoring, Data Management: Bernard Brandewiede and Dr. Katja Krockenberger, AMEDON GmbH, Willy-Brandt-Allee 31c, 23554 Lübeck, Germany - Socioeconomic analyses: Axel Kaiser and Elias Olshausen, Lohfert & Lohfert AG, Rothenbaumchaussee 76, 20148 Hamburg |
| **Contact for Public Queries** | Dr. Miriam Mann, Universitätsmedizin Rostock Versorgungsstrukturen GmbH, Ernst-Heydemann-Str. 8, 18057 Rostock, Germany, email: miriam.mann@med.uni‑rostock.de, phone: +49 451 494 5073 |
| **Contact for Scientific Queries** | - Principle Investigator: Prof. Dr. Christian Schmidt, Ärztlicher Vorstand und Vorstandsvorsitzender, Universitätsmedizin Rostock, Ernst-Heydemann-Str. 8, 18057 Rostock, Germany, email: aev@med.uni‑rostock.de, phone: +49 451 494 5011 - Scientific queries to: Dr. Katja Krockenberger, AMEDON GmbH, Willy-Brandt-Allee 31c, 23554 Lübeck, Germany, email: k.krockenberger@amedon.de, phone: +49 451 38 450226 |
| **Public Title** | CardioCare: Das neue Versorgungsprogramm für Herzpatienten |
| **Scientific Title** | CardioCare MV: A randomized controlled trial for investigating a novel integrated care concept (NICC) for patients suffering from chronic cardiovascular disease |
| **Countries of Recruitment** | Germany, State: Mecklenburg-Vorpommern |
| **Health Conditions** | Atrial fibrillation (EHRA II-IV), heart failure (NYHA II-IV) or treatment resistant hypertension (≥ 3 antihypertensive medicines from different drug classes with SBP > 140/90 mmHg or ≥ 4 antihypertensive medicines from different drug classes) |
| **Interventions** | **Intervention:**  Novel integrated care concept (NICC), consisting of telemedicine (patient receives specific hardware and software), care center as call center, intensive support by call center in case of irregularities in patient data, additional inpatient or outpatient treatment in case of irregularities in patient data as well as guideline therapy.  **Control intervention:**  Standard care will be based on current treatment guidelines of the European Society of Cardiology (ESC) and doctor’s consultation as required. |
| **Key Inclusion and Exclusion Criteria** | **Inclusion criteria:**   - Heart failure (I50, NYHA II-IV) or atrial fibrillation (I48, EHRA II-IV) or resistant hypertension (I10-15, ≥ 3 antihypertensive medicines from different drug classes with SBP > 140/90 mmHg or ≥ 4 antihypertensive medicines from different drug classes). - Member of health insurance companies AOK Nordost or Techniker Krankenkasse (TK). - Inscription to integrated care contract with the health insurance company. - Residence in Mecklenburg-Vorpommern. - Age ≥ 18 years. - Written informed consent.   **Exclusion criteria:**   - Pregnancy, suspected pregnancy or breast-feeding period. - Participation in other clinical trials up to 30 days before inclusion in this trial. - Cognitive deficits. - Chronic kidney disease. |
| **Study Type** | - Interventional - Randomized - Open-label - Parallel group - Multicenter - Randomization: permuted block randomization with variable block length; randomization electronically after complete patient registration using validated software; randomization result will only be transferred after complete electronic patient registration |
| **Date of First Enrollment** | December 04, 2017 |
| **Target Sample Size** | Approximately 2930 in total, 1465 per group. |
| **Recruitment Status** | Ongoing |
| **Primary Outcomes** | - First primary endpoint: composite endpoint consisting of mortality, stroke and myocardial infarction within the 1-year observation period. - Second primary endpoint: number of hospitalizations within the 1-year observation period. - Third primary endpoint: composite endpoint consisting of mortality, stroke, myocardial infarction and cardiovascular decompensation within the 1-year observation period. |
| **Key Secondary Outcomes** | **Efficacy:**   - Separate tests for the outcome measures of the primary endpoint. - Time to event (combined endpoints as well as separate testing for mortality, stroke, myocardial infarction and cardiovascular decompensation). - Number of inpatient days within the 1-year observation period. - Number of inpatient days and number of hospitalizations because of cardiovascular disease within the 1-year observation period. - EuroQol (EQ-5D) at baseline, 6 and 12 months. - NYHA stadium, EHRA stadium, number of antihypertensives, blood pressure at the end of the 1-year observation period. - Treatment cost within the 1-year observation period. - Change in outcome between baseline and follow ups. - Adherence to NICC and its association with outcome.   **Safety:**   - Possible safety events include: mortality, stroke, transitory ischemic attack, myocardial infarction, hospitalization, cardiovascular decompensation, all measured at the end of the 1-year observation period. |
